# Transcriptome analysis of *atad3-*null zebrafish embryos elucidates possible disease mechanisms

**DOI:** 10.1186/s13023-025-03709-0

**Published:** 2025-04-15

**Authors:** Shlomit Ezer, Nathan Ronin, Shira Yanovsky-Dagan, Shahar Rotem-Bamberger, Orli Halstuk, Yair Wexler, Zohar Ben-Moshe, Inbar Plaschkes, Hadar Benyamini, Ann Saada, Adi Inbal, Tamar Harel

**Affiliations:** 1 Department of Genetics, Hadassah Medical Organization, Jerusalem, Israel; 2 Faculty of Medicine, Hebrew University of Jerusalem, Jerusalem, Israel; 3 Department of Medical Neurobiology, Institute for Medical Research-Israel-Canada, Jerusalem, Israel; 4 The Hebrew University-Hadassah Medical School, Jerusalem, Israel; 5 Department of Neurobiology, The George S. Wise Faculty of Life Sciences, Tel-Aviv University, Tel Aviv, Israel; 6 Info-CORE, Bioinformatics Unit of the I-CORE, The Hebrew University of Jerusalem, Jerusalem, Israel; 7 Department of Laboratory Sciences, Hadassah Academic College , Jerusalem, Israel; 8 Department of Genetics, Hadassah-Hebrew University Medical Center, POB 12000, Jerusalem, Israel

**Keywords:** *ATAD3A*, CRISPR/Cas9, Zebrafish knockout model, Transcriptome, RNA-seq, Mitochondria

## Abstract

**Background:**

*ATAD3A*, a nuclear gene encoding the ATAD3A protein, has diverse roles in mitochondrial processes, encompassing mitochondrial dynamics, mitochondrial DNA maintenance, metabolic pathways and inter-organellar interactions. Pathogenic variants in this gene cause neurological diseases in humans with recognizable genotype-phenotype correlations. Yet, gaps in knowledge remain regarding the underlying pathogenesis.

**Methods:**

To further investigate the gene function and its implication in health and disease, we utilized CRISPR/Cas9 genome editing to generate a knockout model of the zebrafish ortholog gene, *atad3*. We characterized the phenotype of the null model, performed mitochondrial and functional tests, and compared the transcriptome of null embryos to their healthy siblings.

**Results:**

Analysis of *atad3*-null zebrafish embryos revealed microcephaly, small eyes, pericardial edema and musculature thinning, closely mirroring the human rare disease phenotype. Larvae exhibited delayed hatching and embryonic lethality by 13 days post-fertilization (dpf). Locomotor activity, ATP content, mitochondrial content, and mitochondrial activity were all reduced in the mutant embryos. Transcriptome analysis at 3 dpf via RNA-sequencing indicated decline in most mitochondrial pathways, accompanied by a global upregulation of cytosolic tRNA synthetases, presumably secondary to mitochondrial stress and possibly endoplasmic reticulum (ER)-stress. Differential expression of select genes was corroborated in fibroblasts from an affected individual.

**Conclusions:**

The *atad3*-null zebrafish model emerges as a reliable representation of human *ATAD3A*-associated disorders, with similarities in differentially expressed pathways and processes. Furthermore, our study underscores mitochondrial dysfunction as the primary underlying pathogenic mechanism in *ATAD3A-*associated disorders and identifies potential readouts for therapeutic studies.

**Supplementary Information:**

The online version contains supplementary material available at 10.1186/s13023-025-03709-0.

## Introduction

*ATAD3A* is a nuclear gene that encodes ATPase family AAA domain-containing 3, a mitochondrial protein functioning as a hexamer spanning the inner and outer mitochondrial membranes. It is crucial to mitochondrial dynamics and influences the structure and function of the mitochondrial inner membrane and cristae [1], causing elongated or fragmented mitochondria when over- or under-expressed, respectively [2, 3]. Mice with a neuron-specific *Atad3* conditional knockout (KO) showed morphological changes in the mitochondria and cristae months prior to onset of neurological symptoms [1]. Additionally, ATAD3A is implicated in mitochondrial DNA (mtDNA) maintenance [4]. It binds mitochondrial transcription factor A (TFAM), facilitating nucleoid trafficking along mitochondria and affecting respiratory complex formation [5]. ATAD3A is also integral to cholesterol metabolism [6]. Loss of ATAD3A results in accumulation of free cholesterol and triglycerides in hepatocytes, promoting nonalcoholic fatty liver disease (NAFLD) [7]. In neurons, ATAD3A oligomerization induces cholesterol accumulation by inhibiting CYP46A1 [8]. Furthermore, ATAD3A is involved in the interaction between endoplasmic reticulum (ER) and mitochondria [9, 10], and is a crucial component in the mitochondria-associated membranes (MAM). It has a role in MAM integrity through interaction with the sigma-1 receptor, and disruptions can cause neurological pathogenesis [11]. Aberrant ATAD3A oligomerization is associated with poor MAM integrity [8]. Elevated *ATAD3A* expression is correlated with poor prognosis in various cancers [12]. In colorectal cancer, it provides chemoresistance by interacting with GRP78, suppressing ER-stress [13]. In breast cancer, it prevents PD-L1 mitochondrial distribution through PINK1 inhibition, conferring tumor resistance [14]. ATAD3A also regulates mitophagy by reducing PINK1 stability [15]. Finally, ATAD3A has a role in the immune cGAS-STING dependent response, as pathogenic variants in this gene cause an upregulation of interferon-stimulated gene (ISG) expression [16].

*ATAD3A* pathogenic variants cause distinct neurological diseases in humans with a predictable genotype-phenotype correlation. At one end of the spectrum are biallelic loss-of-function single nucleotide variants and biallelic deletions mediated by non-allelic homologous recombination (NAHR) between *ATAD3A* and its paralogs *ATAD3B* and *ATAD3C*, which lead to a neonatal-lethal phenotype including severe brain malformations (i.e., simplified gyration patterns, white matter abnormalities and pontocerebellar hypoplasia) and respiratory failure [3, 17, 18]. The reciprocal NAHR-mediated duplication gives rise to a similar lethal phenotype, associated with a dominant-negative mechanism [19, 20]. At the other end of the spectrum is a recurrent dominant, monoallelic *de novo* variant (NM_001170535.3(*ATAD3A*): c.1582 C > T, p.(Arg528Trp)) leading to developmental delay, optic atrophy, cardiomyopathy and peripheral neuropathy via a dominant-negative mechanism [3, 21]. Elsewhere alongst the spectrum are biallelic hypomorphic variants in *ATAD3A*, whose clinical impact correlates with the severity of the mutation as modelled in the *Drosophila* gene *bor* [18].

The zebrafish ortholog gene, *atad3*, shares about 80% similarity to *ATAD3A*, and lacks paralogs. Zebrafish are commonly used as models for many human diseases due to a relatively high degree of genetic resemblance between orthologs and their rapid development. They offer an excellent model for studying mitochondrial diseases due to the well-studied development of their nervous system [22]. The transparent embryos allow for tracking of the phenotype throughout development.

Despite considerable research in the field, a knowledge gap remains regarding the mechanism of disease in humans. In order to contribute to this understanding, and to identify phenotypic and functional readouts in an animal model which can be used to track therapeutic effects in future studies, we generated and characterized *atad3*^*−/−*^ zebrafish at the phenotypic, mitochondrial and transcriptional levels.

## Materials and methods

### *atad3* expression in wild-type (WT) embryos

The research was approved by the local ethics committee (MD-20-16420-1). Guide for the Care and Use of Laboratory Animals guidelines were followed. AB/TL hybrid zebrafish (*Danio rerio*) were used as WT. Adults were maintained according to standard procedures on a 14-h light/10-h dark cycle at 28 °C. Embryos were produced by pair mating and raised at 28.5 °C in egg water (0.3 gr NaCl in 1-liter distilled H_2_O).

RNA was extracted from pools of WT embryos at different developmental stages (namely 6, 12, 24, and 48 hours post-fertilization, hpf). cDNA was prepared using the qScript cDNA Synthesis Kit (Quantabio), and semi-quantitative reverse transcription polymerase chain reaction (RT-PCR) was performed on *atad3* compared to *eef1a1l1* as a housekeeping gene (primers provided in Supplementary Table 1).

Whole-mount in situ hybridization using riboprobes for *atad3* (primers provided in Supplementary Table 1) was performed according to standard protocol (Thisse and Thisse, 2008) on embryos at 48 hpf using DIG RNA Labeling Kits (Roche, Basel, Switzerland).

### Generation of *atad3*-KO in zebrafish by CRISPR/Cas9

CRISPR/Cas9 target sites were designed using the CHOPCHOP tool [23] which identified crRNAs targeted to exons 2 and 3 of *atad3* [guide 3 (gd3) and guide 1 (gd1), respectively, listed in Supplementary Table 1, Additional File 1]. crRNAs were purchased from Integrated DNA Technologies (IDT) and injected separately to zebrafish eggs at the 1-cell stage to generate F0 mosaic embryos. Several F0 embryos were sacrificed to ensure efficacy. The remaining F0 embryos were raised to adulthood. Fin clips were obtained at age 2 months for DNA extraction [24]. Mosaic fish were outcrossed to WT, and the resulting heterozygous F1 fish were genotyped and bred to generate the F2 generation as previously described [25]. Sanger sequencing was used to correlate specific patterns on the acrylamide gel with the resulting indel [25].

### Imaging and image analyses

Images of zebrafish embryos were acquired using Discovery.V8 stereoscope and AxioCam MRc digital camera (Zeiss). Area of head, eye and pericardial sac, length of neurocoel and tail thickness at somites 10 and 20 were measured using ImageJ software. At least 35 embryos/larvae were photographed and measured on four independent occasions: a minimum of 17 mutants, 6 WT and 12 heterozygotes. Numbers vary throughout the days either due to death of embryos or technical issues.

### Mitochondrial assays

Absolute mitochondrial DNA (mtDNA) amount was quantified by qPCR of the mitochondrial genes *mt-nd1*,* mt-nd6* and *mt-cox1* normalized to the nuclear gene *b2m*. Quantification was performed using PerfeCTa SYBR Green FastMix (Quantabio). Primers are listed in Supplementary Table 1, Additional File 1.

ATP level was quantified on heads of zebrafish larvae after snap-freezing in liquid nitrogen by the ATPlite^®^ luciferin-luciferase bioluminescence assay according to the manufacturer’s instructions (Perkin Elmer Waltham MA, USA). Briefly, tissue was homogenized in Mammalian Cell Lysis solution and incubated with Substrate solution. Luminescence was read using Synergy HT BioTek microplate reader and relative luminescence units were normalized to total protein. Total protein was quantified using Pierce™ Detergent Compatible Bradford Assay Reagent (ThermoFisher). Statistical analysis was carried out by a two-tailed, paired T-test.

The activities of respiratory chain complex IV (cytochrome *c* oxidase, COX), complex II + III and succinate dehydrogenase (SDH) were measured by spectrophotometry as previously described, using a double beam Kontron Uvikon 930 BioTek spectrophotometer [26, 27]. Enzymatic activities of COX and complex II + III were normalized to SDH activity and compared to the average activity of siblings. Statistical analysis was carried out by a two-tailed, paired T-test.

### Locomotor activity assays

Mutant larvae and siblings were classified by phenotype at 4 dpf, and then transferred to a DanioVision Observation Chamber (Noldus) at 5 dpf, where the movement of the larvae was recorded for 6 hours (h) and tracked using Noldus Ethovision XT16 [28]. The experimental lighting conditions were as follows: 2 h of white light, followed by 2 h of darkness and 2 more hours of white light (2hrL:2hrD:2hrL). After 5 h, the larvae were exposed to a tap to measure their acoustic startle response [29]. The experiment was repeated with the same larvae the following day (at 6 dpf). The trajectory of each larva was smoothed by LOESS and segmented into periods of progressions and of stops [30]. Responses to the tap and to turning off the light (light-off response, also known as visual motor response [31]) were measured by the distance moved (in cm) in the first 0.5 s after the stimulus.

Comparison of total activity (distance moved), of the tap response intensity and of the light-off response intensity between genotypes was performed using linear mixed models. In all models the genotype, the experimental day (5 dpf or 6 dpf) and the interaction between genotype and day served as fixed effects. Each larva was measured twice, and therefore the larval ID served along with the DanioVision chamber ID as random effects. Square root transformations were required for the models’ assumptions to hold. All post hoc analysis p-values obtained from all models were adjusted simultaneously for multiple comparisons using the BH procedure [32], controlling the false discovery rate at the 0.05 level. Statistical analysis was performed using a designated R code, utilizing the packages *“lmerTest”* [33] and *“emmeans”* [34].

### RNA sequencing (RNA-seq)

Both RNA and DNA were extracted from each 3 dpf embryo using TRIzol Reagent (ThermoFisher). DNA served for genotyping. Respective RNA samples were pooled by genotypes, and analyzed in technical triplicates by RNA-seq. Briefly, libraries were prepared using the KAPPA HyperPrep Plus RNA Library Prep Kit, and were then sequenced on a NovaSeq 6000 sequencing system (Illumina, San Diego, California, USA), generating 160 bp paired-end reads. Raw reads were processed for quality trimming and adaptors removal using fastx_toolkit v0.0.14 and cutadapt v2.10 [35]. The processed reads were aligned to the *Danio rerio* transcriptome and genome version GRCz11 with annotations from Ensembl release 99 using TopHat v2.1.1 [36]. Counts per gene quantification was done with htseq-count v2.01 [37]. Normalization and differential expression analysis were done with the DESeq2 package v 1.36.0 [38]. Pair-wise comparisons were tested with default parameters (Wald test), without applying the independent filtering algorithm. Significance threshold was taken as padj < 0.1. In addition, significant DE genes were further filtered by the log2FoldChange value. This filtering was baseMean-dependent and required a baseMean above 5 and an absolute log2FoldChange higher than 5/sqrt(baseMean) + 0.3 (for highly expressed genes this means a requirement for a fold-change of at least 1.2, while genes with a very low expression would need a 5.8-fold change to pass the filtering).

Whole differential expression data were subjected to gene set enrichment analysis using GSEA [39]. GSEA uses all differential expression data (cutoff independent) to determine whether a priori defined sets of genes show statistically significant, concordant differences between two biological states. We used the following gene sets collections: Gene Ontology Biological Process (GOBP), WikiPathways (WK) and Kyoto Encyclopedia of Genes and Genomes (KEGG), all downloaded from the FishEnrichr database [40]. Differential expression was validated on a group of selected genes by RT-qPCR using PerfeCTa SYBR Green FastMix (Quantabio). Primers are listed in Supplementary Table 1, Additional File 1. Expression was normalized to *gapdh*.

### Cell culture

Fibroblasts from an affected individual (reported in [3] and consented under research protocol H-29697) were maintained in DMEM Dulbecco`s Modified Eagle Media high glucose supplemented with 15% fetal bovine serum, 2mM L-glutamine and 1% penicillin-streptomycin (Biological Industries, Beit Haemek, ISRAEL).

### Statistical analysis

All RT-qPCR experiments were performed in triplicates, and in biological replicates as indicated in the specific method or results. For RT-qPCR and survival assay, statistical analysis was carried out by two-tailed two-sample t-test. For ATP content and enzymatic activity, statistical analysis was carried out by two-tailed, paired t-test, comparing biological replicates of mutants and siblings. For comparison of zebrafish embryo measurements (WT, heterozygotes, and mutants), statistical analysis was carried out by two-way ANOVA test. For hatching analysis, statistical analysis was carried out by chi-squared test. Statistical analysis for locomotor activity was detailed above. *p* values < 0.05 were considered statistically significant for all tests.

## Results

### Expression pattern of *atad3* in zebrafish embryos parallels tissues affected in human disease

*atad3*, the zebrafish ortholog, is highly similar to the human paralogs *ATAD3A* and *ATAD3B* in terms of protein sequence and domains. *ATAD3A* domains from Waters et al., 2023 [41] and Uniprot, as compared to *ATAD3B* and the zebrafish ortholog Atad3, are shown in Supplementary Fig. 1, Additional File 1. To evaluate whether zebrafish embryos would be a proper model for *ATAD3A*-associated disorders, we analyzed the expression levels of RNA extracted from whole embryos at various stages (6, 13, 26, and 48 hpf) by RT-PCR. *atad3* was increasingly expressed through the stages tested (Supplementary Fig. 2A, Additional File 1). Following this, we set to determine by whole mount in situ hybridization the expression pattern in 2 dpf embryos. The strongest levels of detection were identified in specific regions of the brain and in the eyes (Supplementary Fig. 2B, Additional File 1). Since these organs/tissues are also those most affected by *ATAD3A*-associated disorders in humans, we pursued to generate knockout models in zebrafish through CRISPR/Cas9 genome editing.

### CRISPR/Cas9-knockout of *atad3* leads to morphological defects

**Fig. 1:**
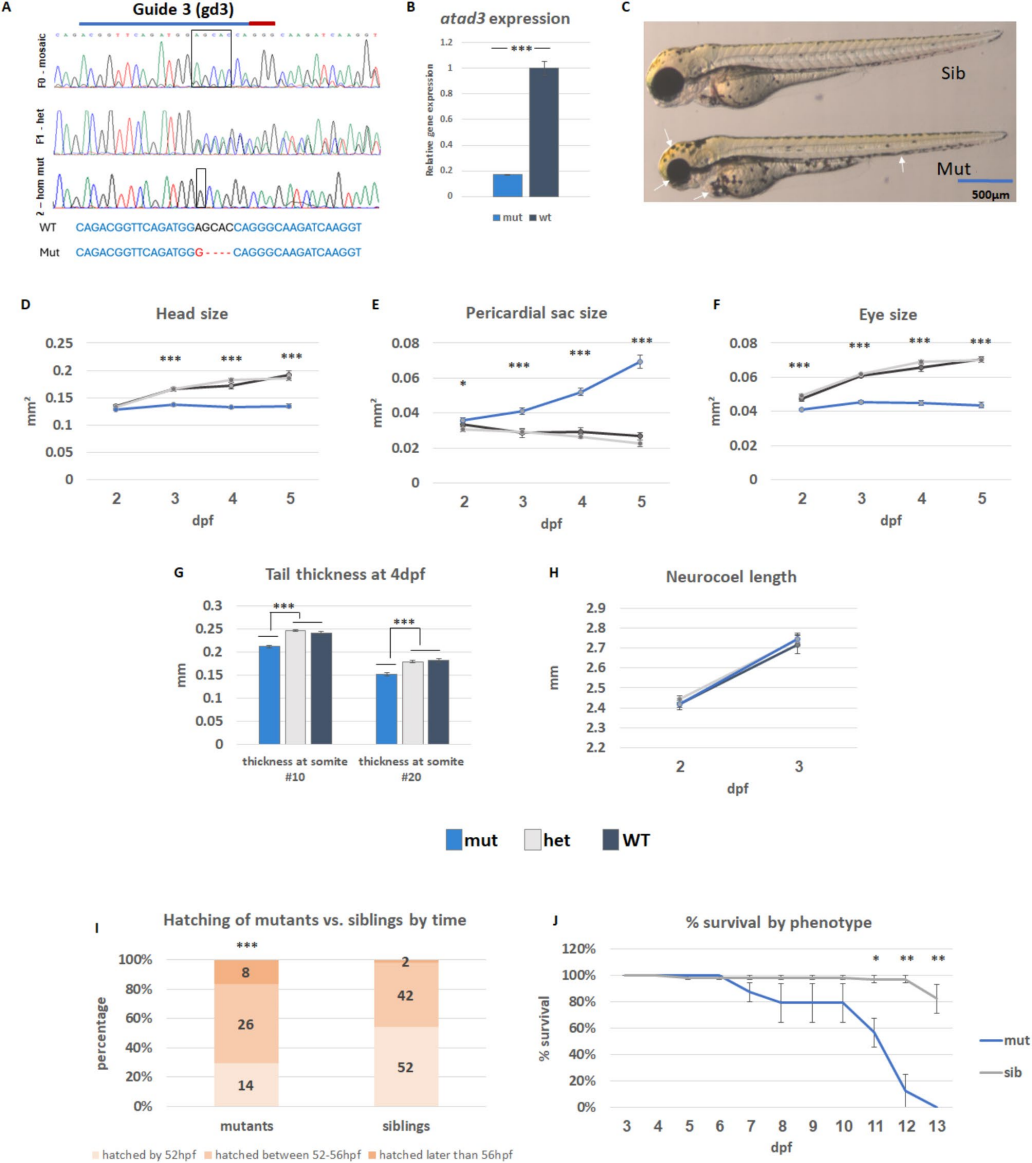
Characterization of *atad3*-null embryos/larvae. (**A**) DNA extracted from mutant embryos confirmed a homozygous out-of-frame deletion (upper panel– F0 generation, mosaic; middle panel– F1 generation, heterozygous; lower panel– F2 homozygous mutant). CRISPR RNA (crRNA) marked by a blue line, PAM sequence marked by a red line. Sequences below show overall 4 bp frameshift caused by 5 bp deletion and 1 bp insertion in the mutant as compared to wild-type (WT). (**B**) Reduced *atad3* expression in mutant embryos. (**C**) Representative images of healthy sibling and mutant embryos (gd3). 35 such embryos (6 WT, 12 heterozygous, 17 mutant) were photographed throughout ages 2–5 dpf and measured (dark grey– WT; light grey– heterozygous, blue– knockout embryos). Mutant embryos showed smaller head size (**D**), pericardial edema (**E**), smaller eyes (**F**), and decreased tail thickness (**G**) despite comparable neurocoel length (H). Error bars indicate standard error of means. (**I**) Hatching time-range for 96 siblings and 48 mutants (*X*^2^ (1, *N* = 144) = 14.9, *p* = 0.000582, indicated above the mutant bar). (**J**) Percent of larvae survival by day, according to phenotype: Mutants in blue (23 total, 7–8 in each biological replicate) and siblings in grey (66 total, 21–23 in each biological replicate). Error bars indicate the standard error of means of three biological replicates. Asterisks represent levels of significance (*p < 0.05, **p < 0.01, ***p < 0.001)

Two CRISPR/Cas9 lines were generated, designated gd1 and gd3. Briefly, the Cas9 endonuclease is guided to a specific genomic location by the designed guide and induces a double-strand break (DSB) in the DNA. This DSB can be repaired by the cellular repair mechanism of non-homologous end joining, which often introduces deletions or insertions. Gd1 fish carried a 14 bp deletion and gd3 fish had a 4 bp deletion in exons 3 and 2 respectively (NM_205703.1) (Fig. 1A and Supplementary Fig. 3A, Additional File 1), in both cases causing a frameshift with a premature termination codon triggering nonsense mediated decay (NMD), as apparent by the 83% reduction (*p* < 0.001) in *atad3* mRNA (Fig. 1B). Mutant embryos could be easily recognized by 3 dpf, and became more distinct as they grew. Interestingly, the mutant phenotype closely correlated with the human phenotype described for *ATAD3A*-related disease. Notable phenotypic features included microcephaly, small eyes, pericardial edema, and thinning of the musculature in homozygous mutants as compared to siblings; for all measurements except for length, *p* < 0.001 from 3 dpf (Fig. 1C). The length of the neurocoel (extending from the head to the tail of the embryo) of the mutant and normal sibling embryos was not significantly different on day 2 nor 3, indicating that the small head and eyes of the mutant embryos were not part of an overall slower development. The swim bladder, which usually inflates starting at 4 dpf, failed to develop. WT and heterozygotes (combined referred to as siblings or sibs) were indistinguishable at any age (Fig. 1D-H). Measurements were performed on gd3 embryos. Gd1 showed a similar phenotype (Supplementary Fig. 3B; Additional File 1).

### *atad3*-null embryos hatch later than their siblings and have reduced survival

The mutant embryos generally hatched later than the siblings, all within normal time range for zebrafish hatching. Among mutants, 29% hatched by the first time point (52 hpf), 54% between the first and second (52–56 hpf) and 17% after the second (> 56 hpf), while of the siblings, 54% hatched by the first time point, 44% between the first and the second and only 2% hatched after the third time point checked, showing a significant difference between mutants and siblings (*X*^2^ (1, *N* = 144) = 14.9, *p* = 0.000582) (Fig. 1I).

Mutant larvae began to exhibit lethality at 7 dpf, and by 13 dpf none of the 23 mutant larvae survived. Conversely, survival rate in the sibling group (consisting of 66 embryos) was close to 100% until 12 dpf, with a statistically significant difference between the groups starting at 11 dpf (*p* = 0.022) and increasing throughout days 12 and 13 (*p* = 0.0027 and *p* = 0.0015, respectively) (Fig. 1J).

### *atad3*-null larvae have decreased mitochondrial DNA content

**Fig. 2:**
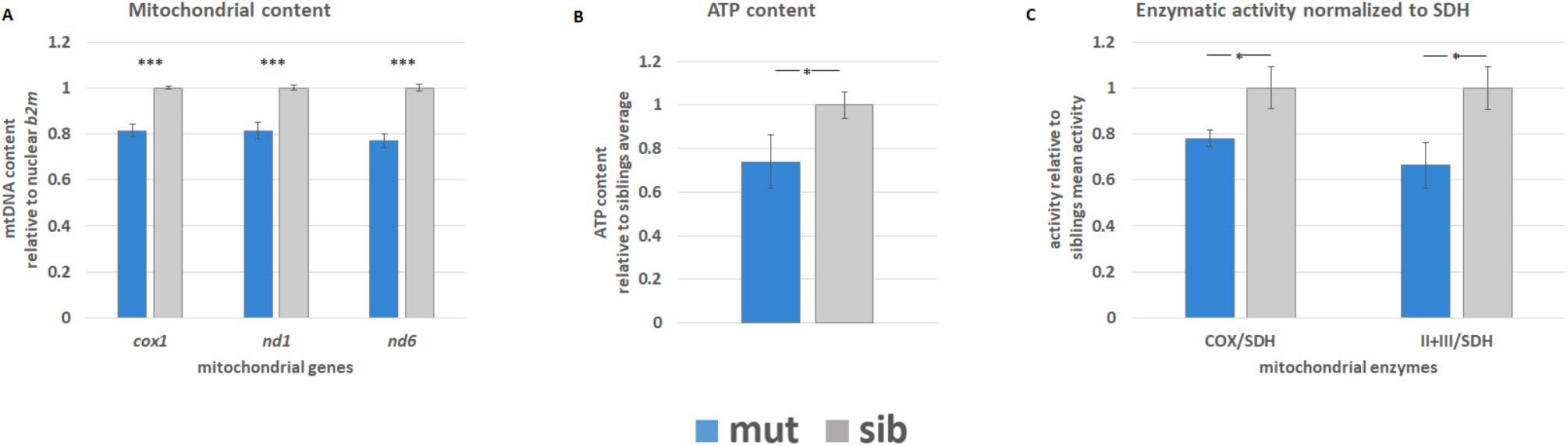
Mitochondrial characterization and ATP content of *atad3*-null larvae. Mutants in blue, siblings in grey. (**A**) mtDNA content comparison between pools of mutant embryos and their healthy siblings (5 dpf); 3 biological replicates of pools (3–8 mutants each, 6–11 siblings each). (**B**) ATP content in the heads of 6 dpf embryos, relative to average sib ATP content measured on the same day; four biological replicates, two from each day. 3 heads in each sample. (**C**) Enzymatic activity of COX and complex II + III normalized to SDH activity relative to the average enzymatic activity of siblings. Measured from 3 biological replicates, 7–9 heads of 5 dpf larvae in each sample. Error bars indicate standard error of means. Asterisks represent levels of significance (*p < 0.05, **p < 0.01, ***p < 0.001)

Previously, pathogenic *atad3* variants in a *Drosophila* model were shown to have decreased mtDNA content, probably due to increased mitophagy [3]. We therefore hypothesized that the overall mitochondrial content in zebrafish null larvae will be reduced. A mtDNA content assay, based on comparison of mitochondrial content normalized to nuclear content by qPCR, revealed a consistent and significant reduction of 19–23% (*p* < 0.001) in mtDNA content in mutant larvae compared to their phenotypically healthy siblings at 5 dpf (Fig. 2A).

### ATP content is decreased in heads of mutant larvae compared to siblings

Based on the results of in situ hybridization which demonstrated that *atad3* expression levels were highest in the head, we determined ATP content in this region. Analysis of mutant 6 dpf larvae relative to the average ATP content in sibling heads collected on the same day revealed a 26% reduction (*p* < 0.05) in ATP content (Fig. 2B).

### COX, complex II + III activities are decreased in heads of mutant larvae compared to siblings

In accordance with high *atad3* expression levels in the head region, we assessed cytochrome *c* oxidase (COX) and mitochondrial complex II + III activity in heads of mutant larvae. Compared to the average siblings’ activity, COX/SDH activity at 5 dpf was reduced to 78%, and complex II + III/SDH activity was reduced to 66% (*p* < 0.05) (Fig. 2C). Notably, SDH is encoded by nuclear genes only, thus our results indicate a decreased activity of the mtDNA encoded respiratory chain complexes in mutant larvae.

### *atad3*-null larvae show decreased locomotor activity

**Fig. 3:**
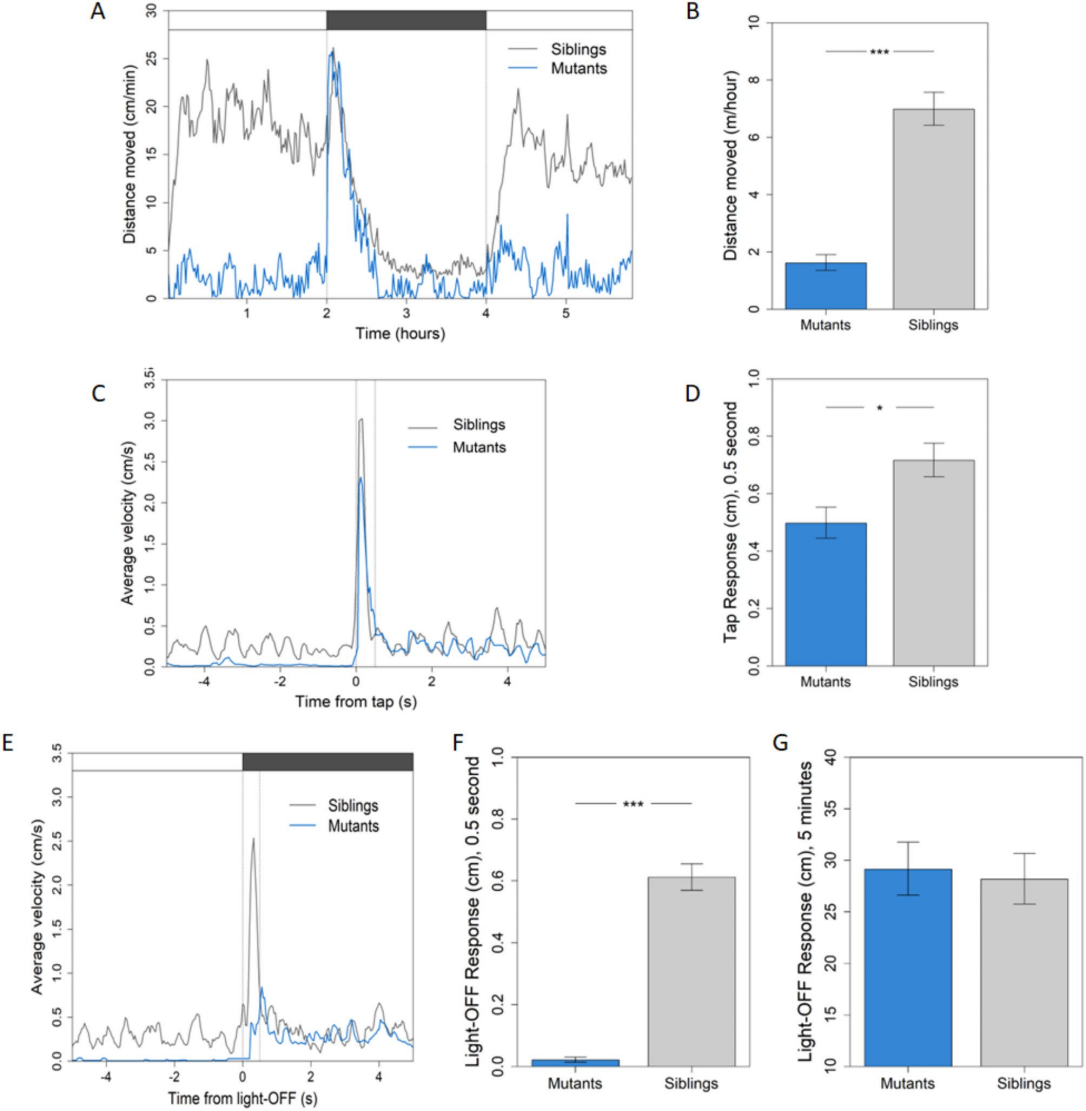
Locomotor activity of *atad3*-null larvae at 5 dpf and 6 dpf is reduced. Mutants in blue, siblings in grey. (**A**) Activity over time at 5 dpf: average distance moved per minute over 6 h (light-dark-light cycle, 2 h each). Upper panel indicates light/dark conditions. (**B**) Mutants demonstrate reduced activity, as measured by average distance moved over 6 h (*p* < 0.001). (**C**) Response to tap in mutants compared to siblings, average of 5 and 6 dpf. Average velocity of mutant larvae immediately after tap (0 s on the X axis) is lower than siblings. (**D**) Average distance moved in the first 0.5 s following tap is 1.4-times lower in mutant larvae compared to siblings (*p* = 0.012). (**E**) Average velocity before and after light-off. (**F**) Initial light-off response: average distance in the first 0.5 s following light-off is reduced in mutants (*p* < 0.001). (**G**) Average distance moved over 5 min after light-off switch shows no difference between mutant and sibling activity (*p* = 0.67). Asterisks represent levels of significance (*p < 0.05, **p < 0.01, ***p < 0.001)

Locomotor assays revealed an overall reduction in movement in the mutant larvae (Fig. 3A). The average distance moved by the siblings was 4.3-fold higher than the mutant larvae (an average of 6.99 m/hour compared to 1.62 m/hour, *p* < 0.001) (Fig. 3B); the difference was greater during light conditions (Fig. 3A). The mutant larvae also had an attenuated immediate reaction to tap (Fig. 3C, D). The average velocity immediately after the tap (0 seconds (s)) was lower in the mutants, and the average distance moved in the 0.5 s following tap in the mutant group was 69% compared to siblings (an average of 0.497 cm and 0.716 cm, respectively; *p* = 0.012). The mutant larvae also had a reduced light-off immediate reaction (Fig. 3E, F) of 3.5% compared to siblings (an average of 0.021 cm compared to 0.612 cm moved in the 0.5 s following light-off, respectively; *p* < 0.001) with a comparable movement rate in the following minutes (*p* = 0.67) (Fig. 3E, G).

### Mutant embryos show significant differential expression of specific biological pathways

**Fig. 4:**
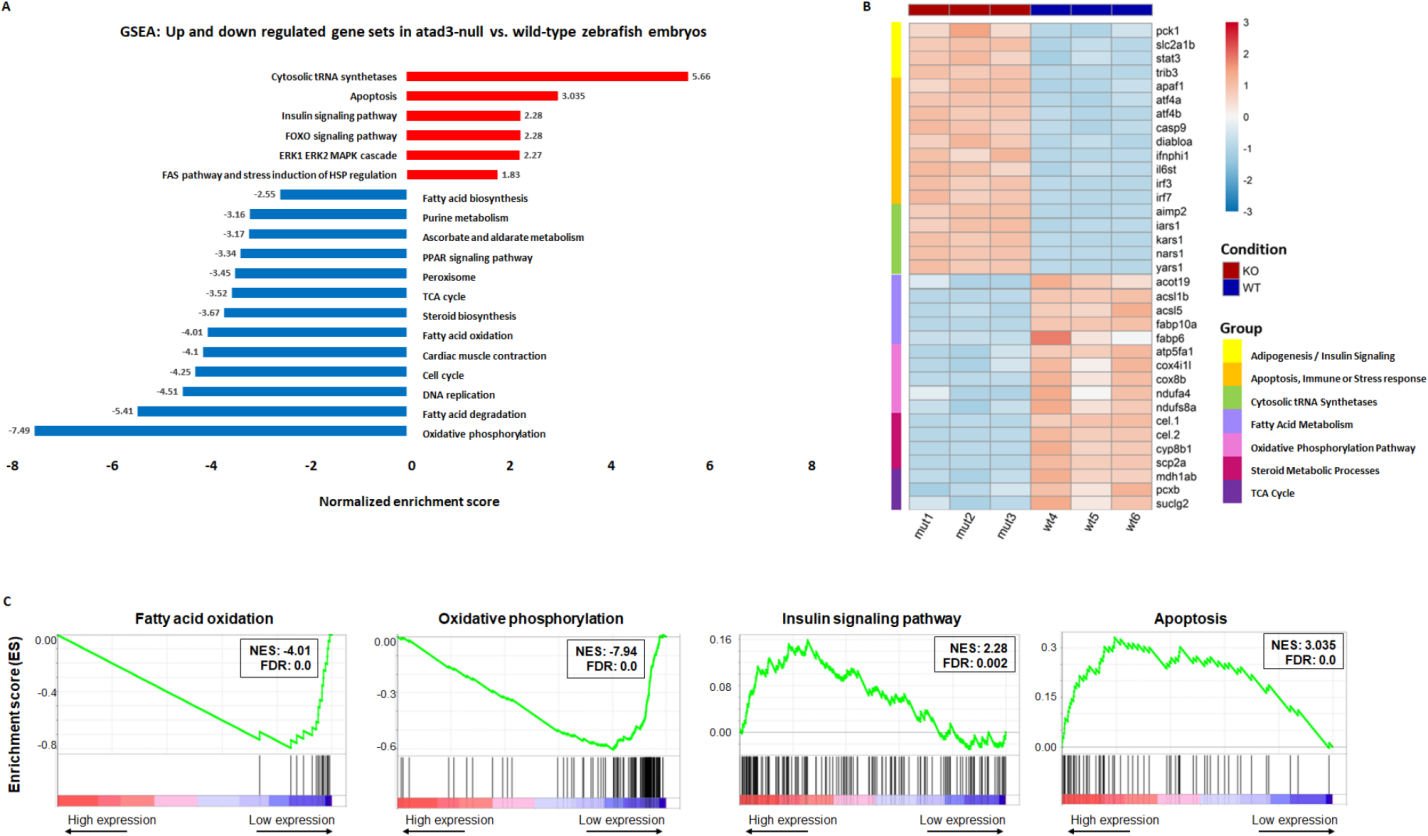
RNA-seq comparing mut 3 dpf embryos to their WT siblings. (**A**) Selected pathways that were downregulated (blue) or upregulated (red) in the *atad3*-null embryos compared to WT. (**B**) Heat-map representing the expression of selected genes from selected gene groups in WT vs. mutant embryo pools. (**C**) Selected GSEA plots showing gene sets that are downregulated or upregulated in KO vs. control. NES: normalized enrichment score; FDR: false discovery rate (corrected *p* value)

To enhance our understanding of the biological pathways perturbed in the mutant embryos, we undertook RNA-seq of homozygous mutant versus their sibling homozygous WT embryos. Siblings were used to minimize batch-specific and timing biases. Extracting DNA and RNA from single embryos enabled individual genotyping followed by pooling of RNA from WT siblings as opposed to all siblings, WT and heterozygotes, providing a significant variance between the two groups (Supplementary Fig. 4; Additional File 1). We identified a total of 2109 genes that were differentially expressed in *atad3-*null zebrafish, consisting of 1261 downregulated and 848 upregulated genes (Supplementary Table 2; Additional File 2). Gene set enrichment analysis (GSEA) was applied to the whole differential expression data, and revealed downregulation of tricarboxylic acid cycle, pyruvate metabolism, oxidative phosphorylation, ascorbate and aldarate metabolism, fatty acid metabolism, biosynthesis, oxidation and degradation, and nucleoid dysfunction– all correlating well with mitochondrial dysfunction. Some pathways were shown to be upregulated, including apoptosis, insulin signaling, adipocytokine signaling, Fas pathway and stress induction of heat shock protein regulation. Innate immune genes linked to interferon signaling were also upregulated. Another gene group that was entirely upregulated was the cytosolic aminoacyl-tRNA synthetases (aaRS genes) (Fig. 4A). These data support the finding of mitochondrial dysfunction related to known *atad3* pathways in the *atad3*-null zebrafish embryos, as well as to mitochondrial stress and possibly to ER stress. Expression of sample genes from selected groups are shown in heat-map (Fig. 4B), and GSEA plots of four selected gene sets are shown in Fig. 4C. GSEA plots of additional gene sets from Fig. 4A can be found in Supplementary Fig. 5; Additional File 1.

**Fig. 5:**
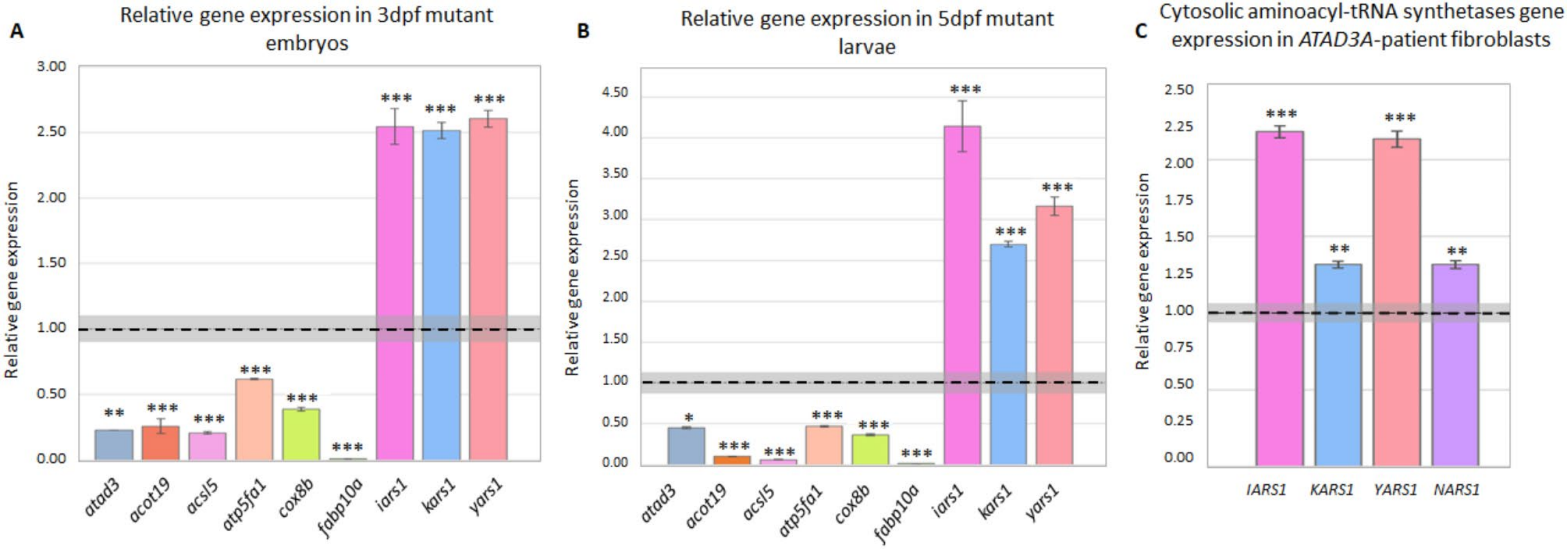
RT-qPCR validations for RNA-seq results. (**A**, **B**) RT-qPCR validation of differential expression by major gene groups: (**A**) at 3dpf. Gene expression in a pool of 20 mutants is normalized to *gapdh* and relative to expression in a pool of 15 siblings, marked by a black dashed line, with siblings average standard error of means of 3 technical replicates in grey. Error bars indicate mutant pool’s standard error of means of 3 technical replicates. (**B**) at 5dpf. Gene expression in a pool of 9 mutants is normalized to *gapdh* and relative to expression in a pool of 9 WT siblings, marked by a black dashed line, with WT average standard error of means of 3 technical replicates in grey. Error bars indicate mutant pool’s standard error of means of 3 technical replicates. (**C**) aaRS genes upregulated in fibroblasts derived from an individual with the monoallelic, dominant negative *ATAD3A* variant. Gene expression is normalized to *GAPDH* and relative to expression in control fibroblasts (marked by a black line), with the average standard error of means of 3 technical replicates in grey. Error bars indicate standard error of means in 3 technical replicates of affected fibroblasts. Asterisks represent levels of significance (*p < 0.05, **p < 0.01, ***p < 0.001).

To determine whether the differential regulation was specific to 3 dpf, or whether it represented a more global trend of *atad3* mutant vs. WT embryos, we chose several genes for validation by RT-qPCR at both 3 dpf and 5 dpf. According to biological pathways, these included cytosolic tRNA synthetases (*kars1*,* yars1*,* lars1*), oxidative phosphorylation (*cox8b*,* atp5fa1*), and fatty acid metabolism (*acsl5*,* acot19*,* fabp10a*) (Fig. 5A). The same trend was confirmed at 5 dpf for all genes tested (Fig. 5B).

### Global rise in cytosolic tRNA synthetases is consistent in fibroblasts derived from an *ATAD3A* patient

To evaluate whether the global rise in cytosolic tRNA synthetases was unique to the zebrafish model or indicative of *ATAD3A* mutant-induced expression changes, RT-qPCR was performed on cDNA derived from fibroblasts of an affected individual with a *de novo* heterozygous *ATAD3A* variant [NM_001170535.3(ATAD3A): c.1582 C > T; p.(Arg528Trp)]. This individual was described in detail in Harel et al., 2016 [3]. The expression of four randomly chosen cytosolic aminoacyl-tRNA synthetases (*YARS1*,* IARS1*,* NARS1*,* KARS1*) was upregulated in the patient compared to control fibroblasts, consistent with the *atad3*-KO model (Fig. 5C).

## Discussion

Through phenotyping, transcriptional profiling and mitochondrial functional assays, we established a zebrafish *atad3*-null model and its relevance for studying gene function and human *ATAD3A*-related disorders. The observed phenotype, affecting the brain, eyes, heart and muscles, is highly consistent and aligns with previous reports in affected individuals [3]. Moreover, the reduction in mitochondrial activity as measured by respiratory chain complexes (complex II + III, COX) activity and a reduction in ATP content, reflect a consistent manifestation of mitochondrial disorders [42]. While our results indicate a reduction in mtDNA content, further investigations are required to differentiate between defective mtDNA replication and reduced mitochondrial mass [43]. Nevertheless, decreased mtDNA content is in accord with decreased mtDNA-dependent activities (II + III and COX) relative to the mtDNA-independent activity of SDH, which is solely encoded in the nuclear genome. With respect to in vivo functional manifestations, the diminished locomotor activity of *atad3*-null larvae, both in resting conditions and after stimulation, indicates a motor and/or neuronal impairment. Survival in the mutant embryos is reduced, similarly to the reported human cases [3, 18–20, 44]. The hatching of both mutants and siblings was within the normal time range of 48–72 hpf [45]. However, hatching of mutants tended to be later within this timeframe. It is unclear whether this observation is indicative of delayed development or muscle weakness [45, 46].

Transcriptional profiling of the *atad3*-null embryos indicated down-regulation of mitochondrial and metabolic pathways known to be connected to human *ATAD3A*, including oxidative phosphorylation, fatty-acids and steroids biosynthesis and metabolism [1, 47]. Major interferon signaling genes were upregulated, consistent with previous reports in human studies [16]. Our study also suggested novel research directions by revealing a specific and global rise in all cytosolic aminoacyl tRNA synthetases (aaRS genes) in *atad3*-null embryos. Other translation pathways and components were not overexpressed, indicating that this rise could not be attributed to a general non-specific upregulation of translation in the mutant embryos. Apart from the main role of these genes in amino acid biosynthesis, aaRS genes are also known to have non-canonical functions unique to the different genes, related to regulation of various cellular processes including immune response, gene expression regulation, RNA splicing, tumorigenesis and more [48–50]. However, the upregulation of all cytosolic aaRS genes suggested a link to their canonical role. Recently, *ATAD3A* was shown to stabilize GRP78 and thus suppress ER stress in colorectal cancer, contributing to chemoresistance [13]. The ER stress response may be mediated by three pathways: IRE1 [51], PERK [52], and ATF6 [53]. We essentially ruled out involvement of the IRE1 pathway in the context of *atad3* deficiency, since *XBP1* splicing was not altered in the raw transcriptome data in *atad3*-null embryos compared to siblings (results not shown) as expected for this pathway [51]. The PERK pathway involves upregulation of *ATF4* [52], which has interestingly been shown to upregulate aaRS genes [54], and was significantly upregulated in the mutant zebrafish. Taken together, this suggests that the ER stress response in the context of *atad3* deficiency may be mediated by this pathway.

A similar upregulation in aaRS genes mediated by ATF4 was previously reported, triggered by mitochondrial stress induced by various effectors on mammalian cells [55]. ATF4 is also active in a non-canonical mitochondrial stress response (mitochondrial unfolded protein response, UPR^mt^) [55]. Nonetheless, we did not observe upregulation of other genes associated with the UPR^mt^ response in the mutant zebrafish. Therefore, we hypothesize that the upregulation of *ATF4* and aaRS genes is mediated by mitochondrial stress and the integrated stress response (IRS), with possible influence from ER stress.

Our transcriptome results also suggest the *atad3-*null zebrafish embryos have a higher level of apoptosis. Furthermore, it shows elevated insulin signaling, adipogenesis and adipocytokine signaling pathways, all pointing towards insulin resistance and related to the known *ATAD3A* effect on fatty-acid metabolism. Mitochondrial dysfunction and specifically ER-mitochondria contact site dysfunction have been shown to lead to insulin resistance [56–58]. Interestingly, a ketogenic diet was previously shown to improve neurological symptoms in patients with *ATAD3A* variants. While one of the variants mentioned (p.Thr84Met, occurring as a biallelic variant in one patient [59] and in trans to a deletion in another patient [60]) was not proven pathogenic, the phenotype of the patients fits the diagnosis of *ATAD3A*-disorder, which was improved by a ketogenic diet [59, 60].

The human paralogs *ATAD3A* and *ATAD3B* are highly similar, yet have some distinct functions. Importantly, *ATAD3A* is the only ubiquitously expressed paralog in human, although *ATAD3B* can have a role in disease [10, 61, 62]. While the lack of *atad3* gene paralogs in zebrafish introduces a potential difference from the human context, the model’s resemblance to human *ATAD3A-*associated diseases suggests its utility in advancing our understanding of these disorders. A limitation of the study is that no rescue studies were conducted. Albeit, we generated models using guides targeting different regions in the gene, and compared the phenotypes. The phenotypes were highly reproducible in both models, suggesting that the observed abnormalities were directly related to *atad3a* knockout and not to off-target effects. Further functional characterization of this model, focusing on the eye, heart and nervous system, is expected to enhance our understanding of the human disorder.

In conclusion, transcriptome analysis of mutant versus WT embryos underscored the importance of mitochondrial dysfunction in the pathogenesis of *ATAD3A-*related disorders and demonstrated an unexpected elevation of cytosolic aaRS genes, secondary to mitochondrial stress and possibly to elevated ER stress. This zebrafish model harnesses the advantages of zebrafish as a model animal and opens avenues for investigating therapeutic interventions and further exploring the underlying mechanisms of *ATAD3A-*related disorders.

## Electronic supplementary material

Below is the link to the electronic supplementary material.

Supplementary Material 1

Supplementary Material 2

## Data Availability

RNAseq data was submitted to Gene Expression Omnibus (GEO): https://www.ncbi.nlm.nih.gov/geo/query/acc.cgi?acc=GSE236968

## Abbreviations

aARS: Aminoacyl-tRNA synthetases
ADP: Adenosine triphosphate
COX: Cytochrome c oxidase
Dpf: Days post-fertilization
DSB: Double-strand break
ER: Endoplasmic reticulum
GOBP: Gene Ontology Biological Process
GSEA: Gene Set Enrichment Analysis
KEGG: Kyoto Encyclopedia of Genes and Genomes
KO: Knockout
MAM: Mitochondrial associated membranes
mtDNA: Mitochondrial DNA
NAFLD: Nonalcoholic fatty liver disease
NAHR: Nonallelic homologous recombination
SDH: Succinate dehydrogenase
WK: WikiPathways
WT: Wild type
