# Supplementary material for: Transcriptome analysis of atad3-null zebrafish embryos elucidates possible disease mechanisms
Source: Orphanet J Rare Dis. 2025 Apr 15;20:181. doi: 10.1186/s13023-025-03709-0 (PMC12001410; doi:10.1186/s13023-025-03709-0)
Supplement: Supplementary file 2 — Supplementary Material 2 [file 13023_2025_3709_MOESM2_ESM.docx]

**Transcriptome analysis of atad3-null zebrafish embryos elucidates possible disease mechanisms**

Shlomit Ezer^1,2^, Nathan Ronin^3^, Shira Yanovsky-Dagan^1^, Shahar Rotem-Bamberger^3^, Orli Halstuk^1,2^, Yair Wexler^4^, Zohar Ben-Moshe^4^, Inbar Plaschkes^5^, Hadar Benyamini^5^, Ann Saada^1,2,6^, Adi Inbal^3^, Tamar Harel^1,2^

1 Department of Genetics, Hadassah Medical Organization, Jerusalem, Israel

2 Faculty of Medicine, Hebrew University of Jerusalem, Israel

3 Department of Medical Neurobiology, Institute for Medical Research - Israel-Canada, The Hebrew University-Hadassah Medical School, Jerusalem, Israel

4 Department of Neurobiology, The George S. Wise Faculty of Life Sciences, Tel-Aviv University, Tel Aviv, Israel

5 Info-CORE, Bioinformatics Unit of the I-CORE, The Hebrew University of Jerusalem, Jerusalem, Israel

6 Department of Laboratory Sciences, Hadassah Academic College Jerusalem

**SUPPLEMENTAL DATA:**

**- Supplementary Figure 1. Clustal Omega alignment of *ATAD3* human paralogs with the zebrafish ortholog *atad3***

**- Supplementary Figure 2. *atad3* expression in zebrafish embryos.**

**- Supplementary Figure 3. Guide 1 (gd1)-targeted CRISPR/Cas9 causes the same effect as gd3-targeted CRISPR/Cas9**

**- Supplementary Figure 4. Principal component analysis (PCA)**

**- Supplementary Figure 5. RNA-seq results: differentially expressed pathways**

**- Supplementary Table 1. Primers and crRNA sequences**

**- Supplementary Table 2. Differentially expressed genes (attached as a separate excel file)**

ATAD3A MSWLFGINKGPKGEGA-GPPPPLPPAQPGAEGGGDRGLGDRPAPKDKWSNFDPTGLERAA 59

ATAD3B MSWLFGVNKGPKGEGA-GPPPPLPPAQPGAEGGGDRGLGDRPAPKDKWSNFDPTGLERAA 59

atad3 MSWLFGLNKGQSGGPPELPPPPAPPAPPGGSG-------AADKPKDKWSNFDPTGLERAA 53

******:*** .* **** *** **..* *****************

ATAD3A KAARELEHSRYAKDALNLAQMQEQTLQLEQQSKLKMRLEALSLLHTLVWAWSLCRAGAVQ 119

ATAD3B KAARELEHSRYAKEALNLAQMQEQTLQLEQQSKL-------------------------- 93

atad3 QAARELDQSRHAKEALDLARMQEQTVQMEHQGKI-------------------------- 87

:*****::**:**:**:**:*****:*:*:*.*:

ATAD3A TQERLSGSASPEQVPAGECCALQEYEAAVEQLKSEQIRAQAEERRKTLSEETRQHQARAQ 179

ATAD3B ----------------------KEYEAAVEQLKSEQIRAQAEERRKTLSEETRQHQARAQ 131

atad3 ----------------------KEYEAAVEQLKGEQIRIQADERRKTLNEETRQHQARAQ 125

:**********.**** **:******.***********

ATAD3A YQDKLARQRYEDQLKQQQLLNEENLRKQEESVQKQEAMRRATVEREMELRHKNEMLRVEA 239

ATAD3B YQDKLARQRYEDQLKQQQLLNEENLRKQEESVQKQEAMRRATVEREMELRHKNEMLRVET 191

atad3 YQDKLARQRYDDQLRQQTLLNEENLRKQEESVQKQEAMRRATIEHEMDLRHKNEMLRVEA 185

**********:***:** ************************:*:**:***********:

ATAD3A EARARAKAERENADIIREQIRLKAAEHRQTVLESIRTAGTLFGEGFRAFVTDWDKVTATV 299

ATAD3B EARARAKAERENADIIREQIRLKASEHRQTVLESIRTAGTLFGEGFRAFVTDRDKVTATV 251

atad3 ESKARARVERENADIIREQIRLKAAEHRQTVLESIRTAGAVFGEGFRAFISDWDKVTATV 245

*::***:.****************:**************::********::* *******

ATAD3A AGLTLLAVGVYSAKNATLVAGRFIEARLGKPSLVRETSRITVLEALRHPIQVSRRLLSRP 359

ATAD3B AGLTLLAVGVYSAKNATAVTGRFIEARLGKPSLVRETSRITVLEALRHPIQVSRRLLSRP 311

atad3 AGLTLLAAGVYSARNATAVAGRYIEARLGKPSLVRETSRFTVAEALKHPIKVVKRLQSKP 305

*******.*****:*** *:**:****************:** ***:***:* :** *:*

ATAD3A QDALEGVVLSPSLEARVRDIAIATRNTKKNRSLYRNILMYGPPGTGKTLFAKKLALHSGM 419

ATAD3B QDVLEGVVLSPSLEARVRDIAIATRNTKKNRGLYRHILLYGPPGTGKTLFAKKLALHSGM 371

atad3 QDALEGVVLSPPLEERVRDIAIATRNTRQNRGLYRNILMYGPPGTGKTLFAKKLAVHSGM 365

**.******** ** ************::**.***:**:****************:****

ATAD3A DYAIMTGGDVAPMGREGVTAMHKLFDWANTSRRGLLLFVDEADAFLRKRATEKISEDLRA 479

ATAD3B DYAIMTGGDVAPMGREGVTAMHKLFDWANTSRRGLLLFMDEADAFLRKRATEEISKDLRA 431

atad3 DYAIMTGGDVAPMGRDGVTAMHKVFDWAATSRRGLLLFVDEADAFLRKRSTEKISEDLRA 425

***************:*******:**** *********:**********:**:**:****

ATAD3A TLNAFLYRTGQHSNKFMLVLASNQPEQFDWAINDRINEMVHFDLPGQEERERLVRMYFDK 539

ATAD3B TLNAFLYHMGQHSNKFMLVLASNLPEQFDCAINSRIDVMVHFDLPQQEERERLVRLHFDN 491

atad3 TLNAFLYRTGEQSNKFMLVLASNQPEQFDWAINDRIDEIVNFMLPGPEERERLVRLYFDR 485

*******: *::*********** ***** ***.**: :*:* ** ********::**.

ATAD3A YVLKPATEGKQRLKLAQFDYGRKCSEVARLTEGMSGREIAQLAVSWQATAYASEDGVLTE 599

ATAD3B CVLKPATEGKRRLKLAQFDYGRKCSEVARLTEGMSGREIAQLAVSWQATAYASKDGVLTE 551

atad3 YVLEPATGGRQRLKLAQFDYGQKCSEIAKRVEGMSGREISKLGVAWQAAAYSSEDGVLSE 545

**:*** *::**********:****:*: .********::*.*:***:**:*:****:*

ATAD3A AMMDTRVQDAVQQHQQKMCWLKAEGPGRGDEPSPS------------------------- 634

ATAD3B AMMDACVQDAVQQYRQKMRWLKAEGPGRGVEHPLSGVQGETLTSWSLATDPSYPCLAGPC 611

atad3 AMIDARVDAAVRQHRQKMDWLHGEGVLDNEGRPVAAETQTKAANMG--FTPPL---KAQE 600

**:*: *: **:*::*** **:.** . :

ATAD3A ------------------------------------- 634

ATAD3B TFRICSWMGTGLCPGPLSPRMSCGGGRPFCPPGHPLL 648

atad3 EL----------RPLQEVPE-----DSQNKHDGTPV- 621

Color codes for *ATAD3A* domains (taken from Unirpot and Waters et al., 2023 [61])


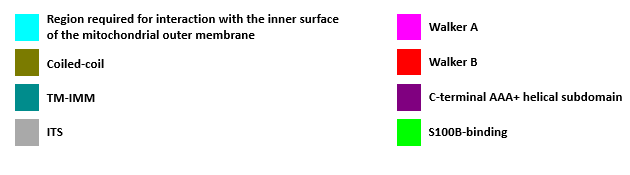


**Figure S1.** Clustal Omega alignment of *ATAD3* human paralogs with the zebrafish ortholog *atad3*


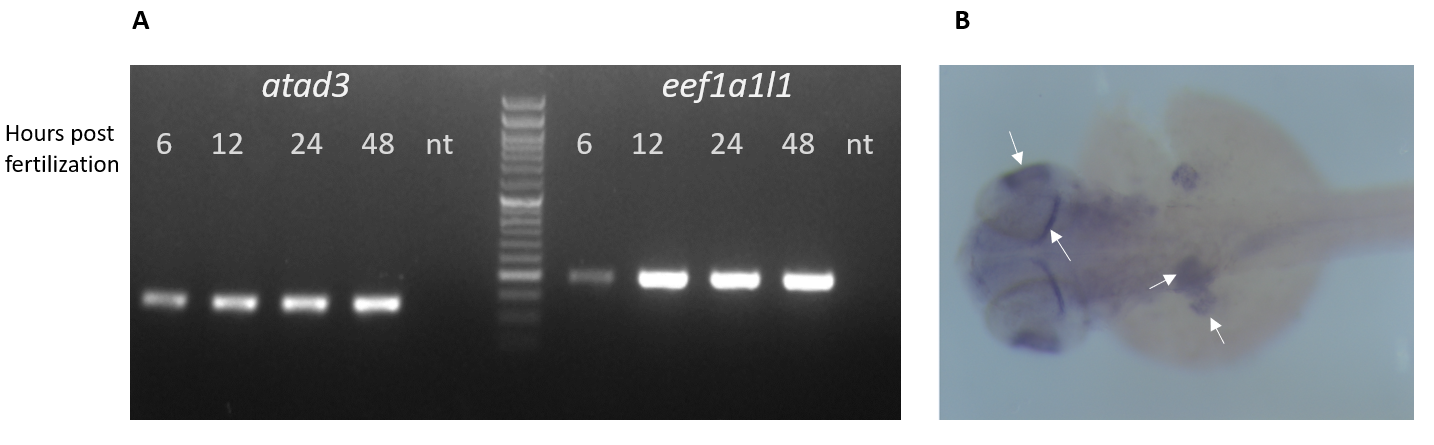


**Figure S2. *atad3* expression in zebrafish embryos.** (A) *atad3* expression in embryos by hours post fertilization (hpf) and nt control (no template) compared to *eef1a1l1* expression (housekeeping gene), RT-PCR; (B) In situ hybridization of *atad3* in a 48hpf zebrafish embryo showing expression (in purple) mainly in the brain, eyes, fin-buds and liver (indicated by white arrows).


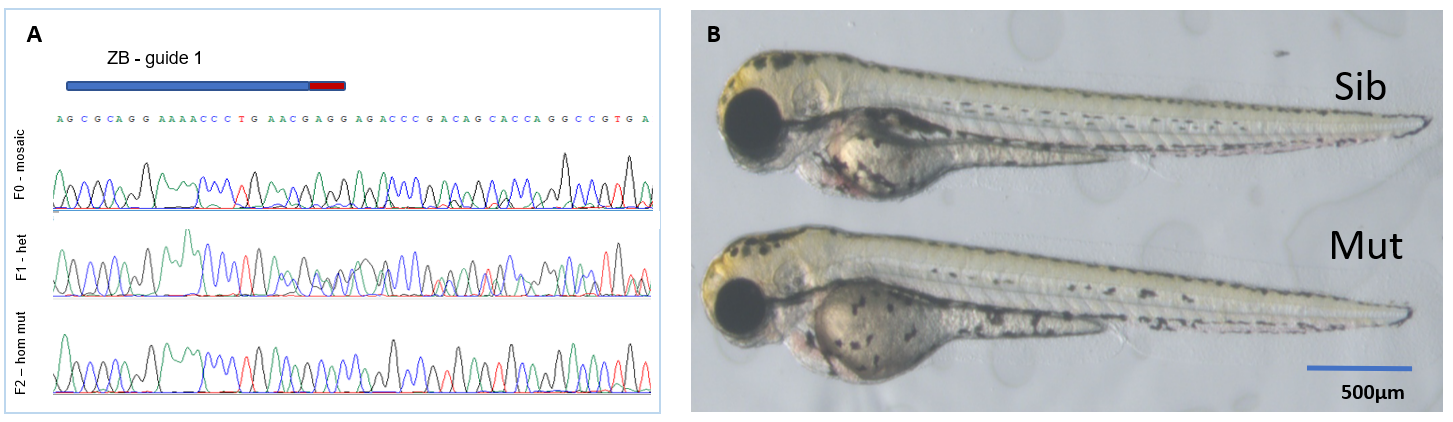
 **Figure S3. Guide 1 (gd1)-targeted CRISPR/cas9 causes the same effect as gd3-targeted CRISPR/cas9.** (A) DNA extracted from mutant embryos was sequenced to confirm a homozygous out-of-frame deletion. Upper panel – F0 generation, mosaic (CRISPR/Cas9-injected fish); middle panel – F1 generation (heterozygous); lower panel – homozygous mutant. CRISPR RNA (crRNA) marked by a blue line, PAM sequence marked by a red line. (B) Sib (either WT or heterozygous) vs. mutant embryo at 3dpf. The mutant embryos show the same phenotype as the gd3 mutants.


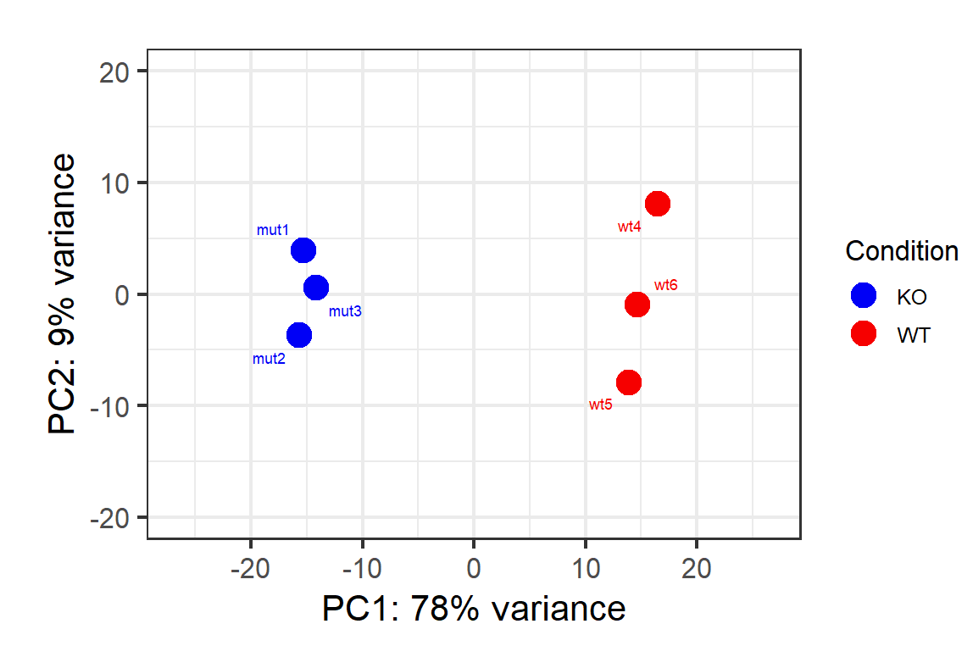


**Figure S4. Principal component analysis (PCA)** indicating global significant difference in RNA expression between WT (in yellow) and mut (in green) embryo pools.


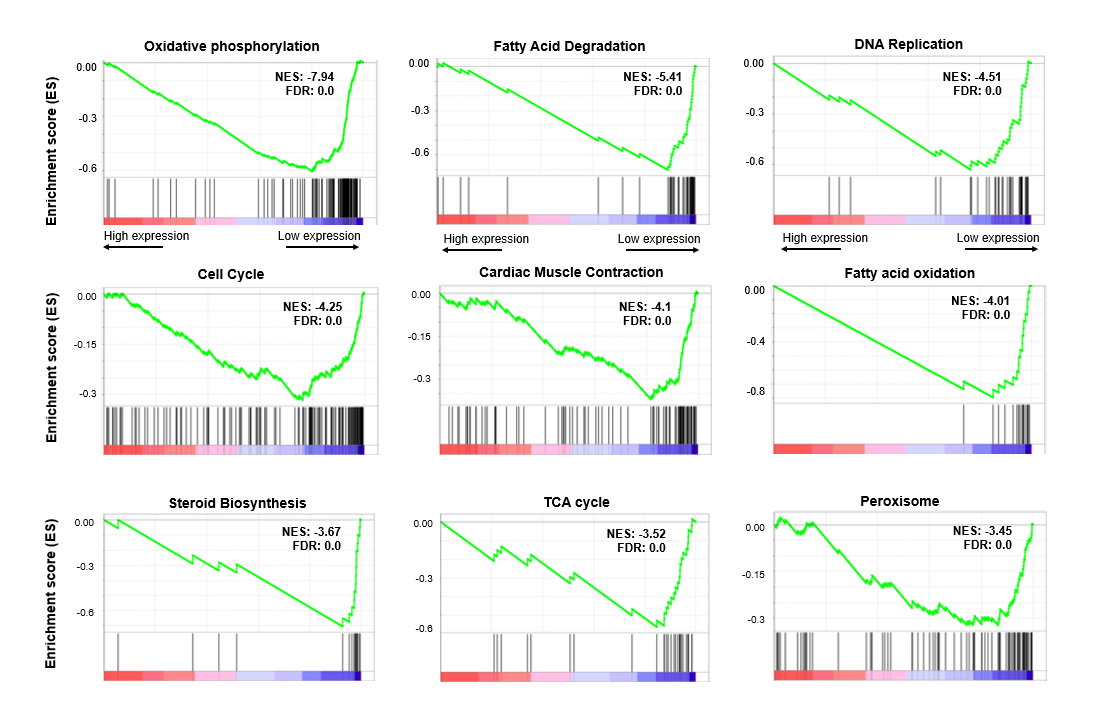

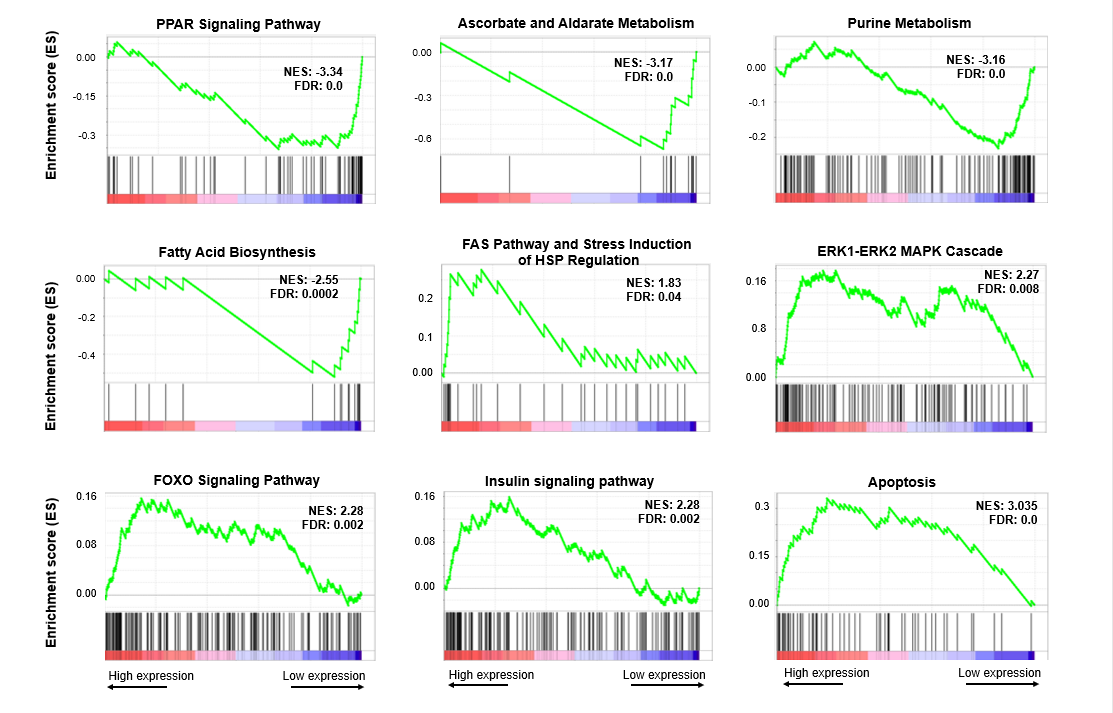


**Figure S5. RNA-seq results: differentially expressed pathways.** Selected GSEA plots showing gene sets that are downregulated or upregulated in KO vs. control. NES: normalized enrichment score; FDR: false discovery rate (corrected *p* value).

**Supplementary Table 1. Primers and crRNA sequences**

| **Primers for semi-quantitative RT-PCR** | | | | |
| --- | --- | --- | --- | --- |
| **Gene** | **F primer** | **R primer** | **Product size** | **Temp** |
| *atad3* | GGACAAATGGAGCAACTTCG | ATCTGAACCGTCTGCTCCTG | 129 | 60 |
| *eef1a1l1* (housekeeping) | ATCTGATCTACAAATGCGGT | GCATCAATAATGGTGACGTAG | 199 | 59 |
| **Primers for sequencing and genotyping** | | | | |
| **Gene** | **F primer** | **R primer** | **Product size** | **Temp** |
| *atad3* (gd3, for acrylamide genotyping) | GTGTTTTACCTTGATCTTGCCC | AGCATGTGTGACCCCTGTG | 112 | 60 |
| *atad3* (gd1, for acrylamide genotyping) | CAGCTGAAGGGTGAGCAGAT | AACAGTGTCCTGCACTCACG | 100 | 60 |
| *atad3* (gd3, for sequencing) | GTGTTTTACCTTGATCTTGCCC | AGGCGAACTCACACTCTGCT | 168 | 60 |
| *atad3* (gd1, for sequencing) | AACAGTGTCCTGCACTCACG | CAGGAGTACGAGGCAGCAGT | 124 | 60 |
| **Primers for mitochondrial content (qPCR)** | | | | |
| **Gene** | **F primer** | **R primer** | **Product size** | **Temp** |
| *b2m* | CGCCTGAAAACTACGTTCTACAC | ACTTTCGGAGTGGCTGAAAA | 140 | 60 |
| *mt-nd1* | AGCCTACGCCGTACCAGTATT | GTTTCACGCCATCAGCTACTG | 143 | 60 |
| *mt-cox1* | TGGAAACTGACTTGTGCCACT | TCATCCTGTTCCAGCTCCTG | 145 | 60 |
| *mt-nd6* | AGTAGTTGTGGCAGGGGTTG | CTTCAGGAAAAGGCTCGGCT | 146 | 60 |
| **RT-qPCR primers** | | | | |
| **Gene** | **F primer** | **R primer** | **Product size** | **temp** |
| *gapdh* (housekeeping) | CGCTGGCATCTCCCTCAA | TCAGCAACACGATGGCTGTAG | 85 | 60 |
| *atad3* | GGACAAATGGAGCAACTTCG | ATCTGAACCGTCTGCTCCTG | 129 | 60 |
| *kars1* | GTTTCCACCTCCTGACACCT | AAGAAGTCGAGCAGTTGTCCT | 112 | 59 |
| *iars1* | CCATCTGGCATATTCACTCCGG | CCGTTCTCCTTCAGCCACTT | 95 | 60 |
| *yars1* | GCTCAGTTTGGTGGTGTGGA | TCAAGTGGCTACGTTTGGTGT | 91 | 60 |
| *sars1* | TCAGCCAGTTTGACGAGGAG | CGATTGGCTGCTCTGATGTG | 111 | 60 |
| *cox8b* | GAGGGCTGCTATGAGACACC | TCACAAACATGACCGACAATGC | 113 | 60 |
| *atp5fa1* | GTCTGTCTGTGTCCCGTGTC | AGCAGCAACCTCACGATACT | 104 | 60 |
| *acsl5* | TGCTCTTTCACCATACCAGGAG | CACCATTGGATGCAAAGTAGTCC | 115 | 60 |
| *acot19* | TGAAACTAAACCCGGTCGCA | GAGAGGAGTGCGATGCTTGA | 115 | 60 |
| *fabp10a* | GCAGGTTTACGCTCAGGAGA | GCTGGATTTCTGTCACTGGC | 107 | 60 |
| **Primers for RNA probes** | | | | |
|  | **F primer** | **R primer** |  |  |
| *atad3* - sense | AATTAACCCTCACTAAAGGG | TAATACGACTCACTATAGGG |  |  |
| *atad3* - antisense | GCGTGACACTCGTGTAGTGG | AGACTCCAGCACGGTCTGTC |  |  |
| **crRNA** | | | | |
| **crRNA target** | **crRNA sequence (PAM in bold)** |  |  |  |
| *atad3* guide 1 | GCGCAGGAAAACCCTGAACG **AGG** |  |  |  |
| *atad3* guide3 | ACGGTTCAGATGGAGCACCA **GGG** |  |  |  |
